# Phase Variance Analysis for Identifying Heterogeneous Conduction Regions in Persistent Atrial Fibrillation: Impact of Recording Duration

**DOI:** 10.1002/joa3.70246

**Published:** 2025-12-09

**Authors:** Hiroshi Seno, Yilin Wang, Toshiya Kojima, Tsukasa Oshima, Kenichiro Yamagata, Masatoshi Yamazaki, Ichiro Sakuma, Katsuhito Fujiu, Naoki Tomii

**Affiliations:** ^1^ Research Center for Advanced Science and Technology The University of Tokyo Bunkyo‐ku Japan; ^2^ Graduate School of Engineering The University of Tokyo Bunkyo‐ku Japan; ^3^ Department of Cardiovascular Medicine The University of Tokyo Hospital Bunkyo‐ku Japan; ^4^ Department of Cardiovascular Medicine Japanese Red Cross Medical Center Tokyo Japan; ^5^ Department of Cardiology Nagano Hospital Nagano Japan; ^6^ Research Institute for Science and Technology Tokyo Denki University Adachi‐ku Japan

**Keywords:** atrial fibrillation, reproducibility of results, treatment outcome

## Abstract

**Background:**

Phase variance analysis, which quantifies the spatial heterogeneity of local activation, has been proposed as a method to characterize complex conduction patterns in atrial fibrillation (AF). However, the influence of recording duration on its reliability remains unclear. This study aimed to determine how recording duration affects the accuracy and reproducibility of identifying heterogeneous conduction regions by phase variance analysis.

**Methods:**

Intracardiac electrogram recordings (30 s) were obtained from 16 left atrial sites in 5 patients with persistent AF. A reference phase variance map (REF) was generated by averaging instantaneous phase variance maps over the full 30‐s dataset to represent the frequency of local conduction heterogeneity. Thirteen recording durations (0.01–25 s) were defined as experimental conditions. For each duration, 100 randomly selected segments were extracted, and estimated phase variance maps (ESTs) were computed using the same temporal averaging method. Accuracy was evaluated by calculating the structural similarity index measure (SSIM) between ESTs and REF, while reproducibility was assessed using pixel‐wise standard deviations across subsamplings.

**Results:**

Longer recording durations produced higher SSIM values and lower standard deviations. With durations ≥ 10 s, SSIM consistently exceeded 0.95, including outliers, and standard deviations fell below 0.015, indicating accurate and stable estimation of complex conduction patterns.

**Conclusions:**

Adequate recording duration enables accurate and reproducible estimation of heterogeneous conduction regions using phase variance analysis, even in persistent AF with complex conduction patterns. This method may support optimal ablation target selection and improve treatment outcomes.

## Graphical abstract

Phase variance analysis applied to inferred membrane potentials enables high‐resolution mapping for identifying heterogeneous conduction regions in persistent atrial fibrillation. This study reveals that a 10‐s recording duration is sufficient to achieve stable and reliable characterization of local activation heterogeneity, supporting its feasibility for future substrate‐guided ablation strategies.

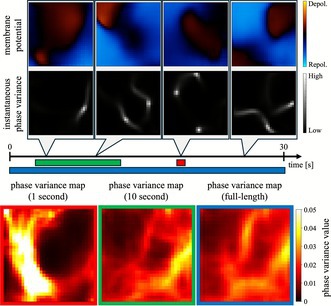

## 1 Introduction

Atrial fibrillation (AF) is a major risk factor for heart failure and stroke, and its prevalence continues to rise worldwide [1, 2]. Catheter ablation, which eliminates abnormal conduction pathways to restore normal sinus rhythm, has become an established curative therapy. Pulmonary vein isolation is highly effective for paroxysmal AF [3, 4], but recurrence rates remain high in nonparoxysmal AF (non‐PAF), and long‐term outcomes are suboptimal [5]. One contributing factor is the absence of reliable methods to accurately visualize and quantify atrial arrhythmogenic substrates, which guide ablation strategies.

To overcome this limitation, several techniques have been developed to characterize arrhythmogenic regions using intraoperative catheter electrograms (ECGs). Common approaches include identification of low‐voltage areas (LVA) [6] and complex fractionated atrial electrograms (CFAE) [7]. Although these indices provide a straightforward means of characterizing conduction abnormalities, multicenter clinical trials of CFAE‐guided ablation have demonstrated limited improvement in outcomes for persistent AF [8].

As an alternative, advanced mapping techniques have been developed to visualize atrial conduction patterns in greater detail using ECGs recorded from mapping catheters. Focal impulse and rotor modulation (FIRM) mapping [9], which applies basket catheter signals to estimate global atrial conduction, was an early pioneering method. More recently, high‐density electrode catheters have enabled more precise assessment of local conduction [10, 11, 12]. These approaches represent important progress in the assessment of arrhythmogenic regions. However, in non‐PAF, where activation patterns are inherently nonstationary and complex, achieving accurate and high‐resolution visualization of conduction patterns remains a significant challenge.

To address these challenges, we recently developed a novel mapping approach, DEAP Mapping, which applies deep learning to estimate membrane potentials from catheter ECGs [13]. The model was trained entirely on in silico datasets generated using a cardiac electrophysiology simulator, enabling supervised learning with full ground‐truth membrane potential information. Its predictive performance was subsequently validated in ex vivo porcine heart experiments, where DEAP Mapping successfully reconstructed membrane potential dynamics with an accuracy comparable to optical mapping. Furthermore, when phase variance analysis [14] was applied to the reconstructed membrane potential movies, it enabled high‐resolution quantitative identification of heterogeneous excitation regions. This combination demonstrated the potential to assess conduction heterogeneity in a manner that conventional methods could not achieve. However, validation of this approach has so far been restricted to ex vivo animal models of acute AF, leaving its applicability in clinical AF uncertain. In particular, non‐PAF is characterized by highly nonperiodic and complex activation patterns, which may necessitate longer ECG recordings for accurate estimation of conduction heterogeneities. Yet, the optimal recording duration required for reliable phase variance analysis remains undetermined.

In this study, we applied DEAP Mapping combined with phase variance analysis to intraoperative catheter ECGs obtained from patients with persistent AF. We then quantitatively evaluated how recording duration influences the accuracy and reproducibility of identifying heterogeneous conduction regions.

## 2 Methods

### 2.1 Participants

We retrospectively analyzed data from five patients with persistent AF who underwent catheter ablation at The University of Tokyo Hospital. The patients exhibited diverse clinical backgrounds: mean age was 58.8 ± 14.1 years (range: 42–74), BMI was 23.1 ± 5.3 (range: 18.4–30.9), duration of persistent AF was 30.6 ± 50.1 months (range: 4–120), left atrial diameter (LA diameter) was 39.0 ± 8.0 mm (range: 25–45), and the cohort included 4 males and 1 female. This study was conducted in accordance with the Declaration of Helsinki and was approved by the institutional review board of The University of Tokyo Hospital (Approval No. 2023232NI).

### 2.2 Data Acquisition

**FIGURE 1:**
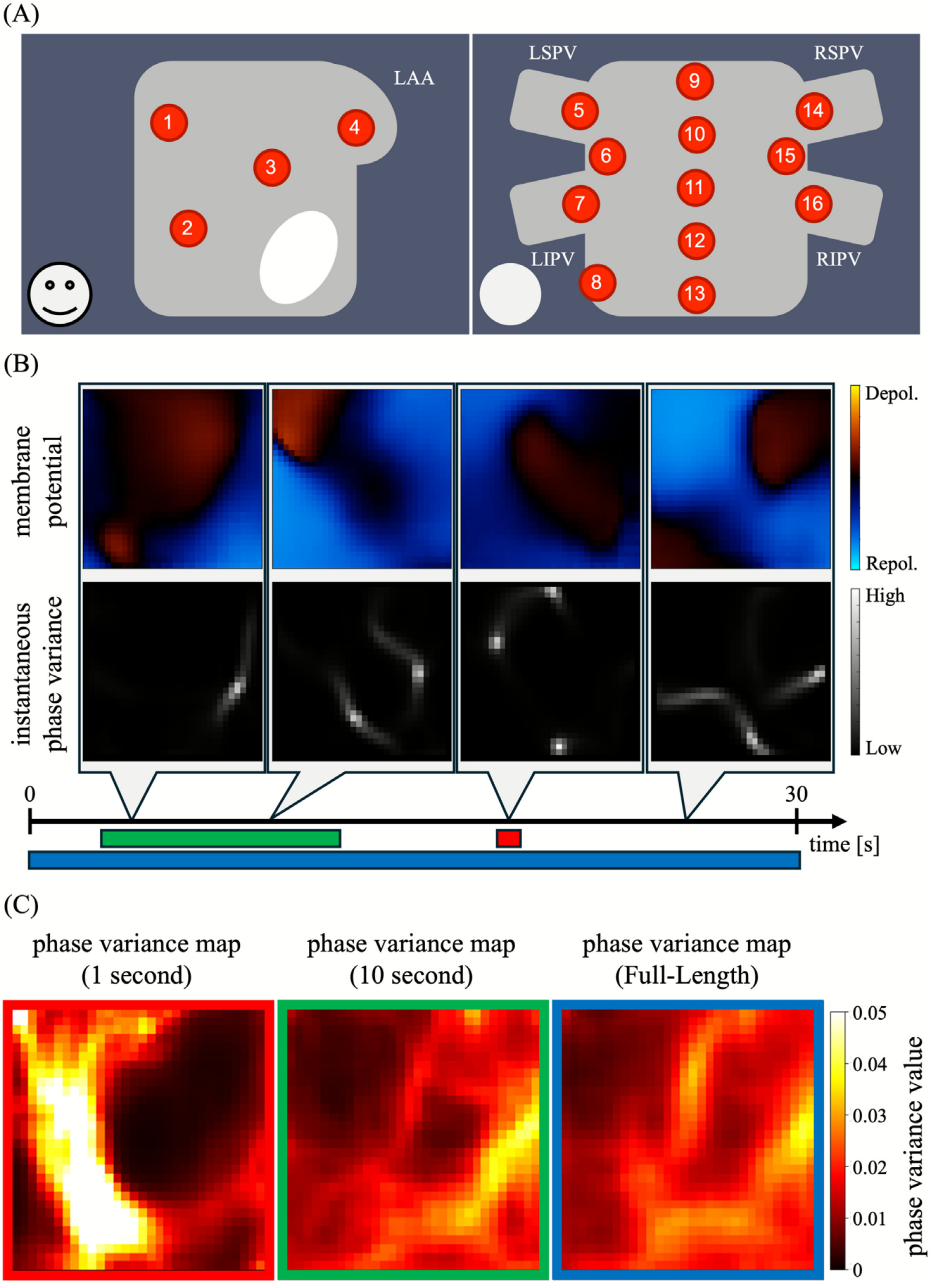
Assessment of conduction heterogeneity using phase variance analysis. (A) Recording sites within the left atrium. Unipolar electrograms were acquired at 16 left atrial locations using a mapping catheter. LAA, left atrial appendage; LIPV, left inferior pulmonary vein; LSPV, left superior pulmonary vein; RIPV, right inferior pulmonary vein; RSPV, right superior pulmonary vein. (B) Representative examples of membrane potential estimations and the corresponding instantaneous phase variance maps. Areas of high phase variance indicate regions where conduction delay and/or block is likely to occur. The lower bar shows the analysis intervals displayed in panel C. (C) Phase variance maps (PV maps) generated with different analysis durations. Left, 1 s (red box); middle, 10 s (green box); right, entire 30 s (reference map, blue box). Longer analysis durations yielded PV maps that more closely resemble the reference map.

Intracardiac ECGs were obtained from 16 left atrial sites during ongoing AF before ablation (Figure 1A). Recordings were acquired using a PENTARAY catheter (Biosense Webster, USA) and a polygraph system (RMC‐5000, Nihon Kohden, Japan) at a sampling rate of 1 kHz. At each site, ECGs were recorded for 30 s.

### 2.3 Evaluation of Heterogeneous Conduction Regions With Phase Variance Analysis

High‐resolution conduction patterns were estimated using DEAP Mapping [13], which infers the spatiotemporal distribution of membrane potentials from intracardiac ECGs. Phase maps were derived from the inferred membrane potentials with the Hilbert transform. At each time point, phase variance analysis was applied to the phase map to quantify spatial heterogeneity of local activation, resulting in an instantaneous phase variance map. To quantify conduction heterogeneity, the first and last 250 ms of data were excluded to minimize edge artifacts, and a phase variance map (PV map) was obtained by averaging the remaining 29 500 instantaneous phase variance maps over time. This time‐averaged map was defined as the reference PV map, representing regions where abnormal conduction (e.g., conduction delays and/or blocks) occurred consistently during the recording.

### 2.4 Evaluation Protocol

To examine how analysis duration influences the accuracy and reproducibility of identifying heterogeneous conduction regions, we conducted the following procedure: From the total 29 500 instantaneous phase variance maps, segments of *T* frames corresponding to different durations were randomly extracted. For each segment, the time‐averaged map was computed as the estimated PV map based on *T* ms of data. Thirteen durations were tested (0.01, 0.02, 0.05, 0.1, 0.2, 0.5, 1, 2, 5, 10, 15, 20, and 25 s), and for each, 100 independent random samples were generated.

Mapping accuracy was assessed by calculating the structural similarity index measure (SSIM) between each estimated PV map and the reference PV map, and SSIM distributions were analyzed for each duration. Mapping reproducibility was assessed by calculating the pixel‐wise standard deviation of estimated PV maps across the 100 samples for each duration, providing a quantitative measure of variability.

## 3 Results

### 3.1 Assessment of Conduction Heterogeneity Using Phase Variance Analysis

Representative assessment results of conduction heterogeneities using phase variance analysis are shown in Figure 1B,C. Figure 1B illustrates the spatial distribution of inferred membrane potentials (top row) and the corresponding instantaneous phase variance maps (bottom row) at four representative time points. Diverse conduction patterns were observed during the 30‐s recording. Localized areas of high phase variance appeared in regions with conduction delay and/or block. The horizontal bar at the bottom denotes the analysis intervals displayed in Figure 1C. Figure 1C shows estimated PV maps generated with different analysis durations. In the 1‐s window (red box), a high‐variance region appeared on the left side, differing markedly from the reference PV map (blue box) generated from the full recording. In contrast, the 10‐s window (green box) produced a PV map more consistent with the reference, suggesting that longer analysis durations yield more stable estimations.

### 3.2 Qualitative Evaluation of Heterogeneous Conduction Estimation

**FIGURE 2:**
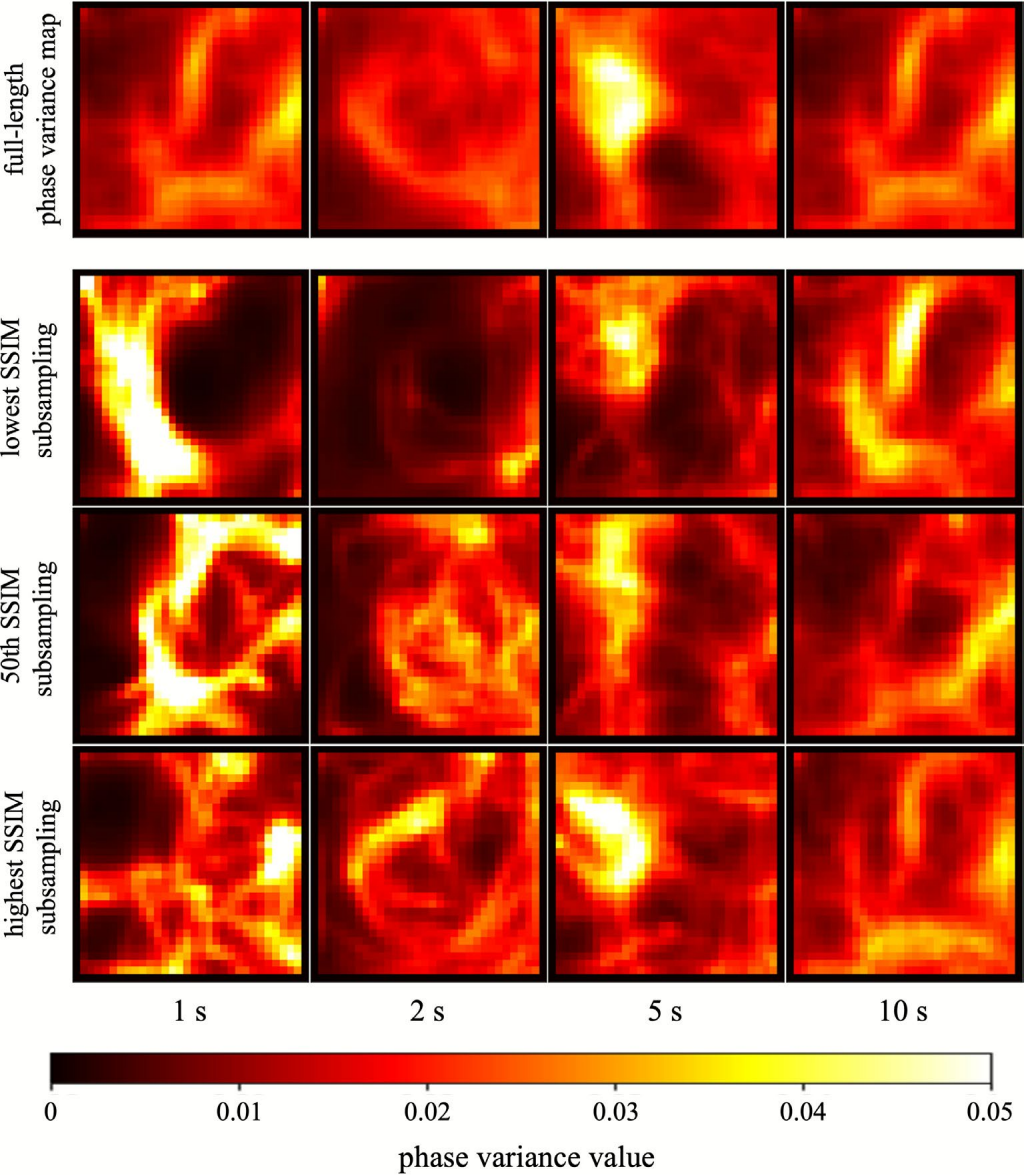
Examples of heterogeneous conduction estimation with different analysis durations. Phase variance maps (PV maps) estimated with analysis durations of 1, 2, 5, and 10 s are shown in the four columns. The top row shows the reference PV map generated from the entire 30‐s recording. The other rows show subsampling results ranked 1st, 50th, and 100th according to structural similarity index measure (SSIM) with the reference map after 100 subsamplings for each duration. Shorter analysis durations exhibited substantial variability in the distribution of high phase variance regions across subsamplings, whereas longer durations yielded PV maps that more closely resembled the reference map.

Figure 2 illustrates how the estimated conduction heterogeneity changes across different analysis durations. The four columns represent analysis windows of 1, 2, 5, and 10 s. The top row shows the reference PV map generated using the full phase dataset, and the three rows below display examples of the 1st, 50th, and 100th ranked maps based on SSIM compared with the reference map from 100 subsampled analyses.

In the top‐ranked subsampling of each duration, all windows produced reasonably accurate representations of the heterogeneous conduction, though minor differences were noted. However, variability across subsamplings was notably greater for shorter durations, particularly 1 and 2 s. In these cases, the spatial locations and shapes of high‐variance regions differed substantially between the best‐ and worst‐ranked samples. As the analysis duration increased, variability decreased, and the spatial structures of high‐variance regions became more consistent across subsamplings. Notably, in the 10‐s analysis, even the 50th‐ and 100th‐ranked subsamples closely resembled the reference map, indicating that longer recording durations contribute to greater stability in heterogeneous conduction estimation.

### 3.3 Quantitative Evaluation of Heterogeneous Conduction Estimation

**FIGURE 3:**
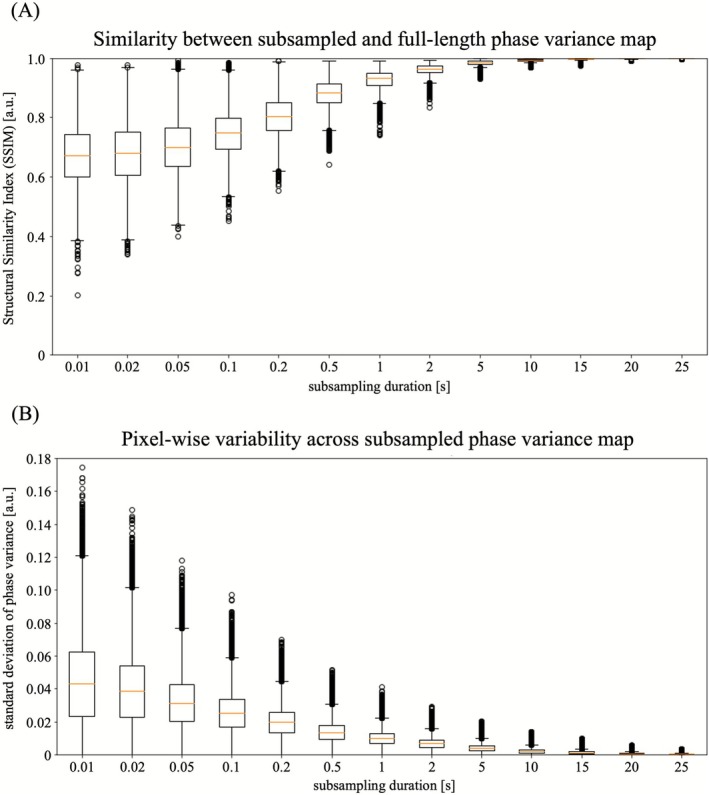
Quantitative evaluation of heterogeneous conduction estimation across different analysis durations. (A) Distribution of structural similarity index measure (SSIM) values between the reference phase variance map (PV map) generated from the entire 30‐s recording and 100 subsampled analyses at each duration. Longer analysis durations yielded higher SSIM values with reduced variability. Notably, for durations ≥ 10 s, SSIM values consistently exceeded 0.95, even when considering outliers. (B) Distribution of pixel‐wise standard deviation of phase variance indices across 100 subsamplings for each analysis duration. The standard deviation decreased with increasing duration. For durations ≥ 5 s, nearly all samples (excluding outliers) converged below 0.01, and for durations ≥ 10 s, values including outliers converged below 0.015.

Figure 3 summarizes the quantitative evaluation of accuracy and reproducibility in estimating heterogeneous conductions.

Figure 3A shows the distribution of SSIM values between the reference PV map and 100 subsampled maps for each analysis duration. SSIM values increased consistently with longer durations, and the spread of the distributions narrowed. Notably, for durations of 2 s or more, the median SSIM exceeded 0.95. For durations of 10 s or more, all 100 subsampled SSIM values were greater than 0.95, including outliers.

Figure 3B shows the pixel‐wise standard deviation of estimated PV maps across the 100 subsamplings. Variability decreased as the analysis duration increased, with most values falling below 0.01 at durations of 5 s or longer (excluding outliers). The maximum standard deviation, including outliers, also declined with longer durations, reaching ~0.015 at 10 s and ~0.01 at 15 s.

## 4 Discussion

### 4.1 Validity of Phase Variance Analysis for Estimating Heterogeneous Conductions

In this study, we applied phase variance analysis to visualize heterogeneous conductions in patients with persistent AF. As shown in Figure 1B, instantaneous phase variance maps quantitatively identified localized regions of abnormal excitation, including conduction delay and block. The PV map represents the spatial frequency of abnormal excitations, providing a stable depiction of heterogeneous conductions. Unlike conventional mapping methods, phase variance analysis enables high‐resolution quantitative evaluation of conduction heterogeneities even under nonperiodic and complex conduction patterns. This capability is particularly valuable during catheter ablation, where identifying regions with frequent abnormal excitations may inform optimal ablation strategies. Accordingly, PV maps derived from phase variance analysis hold promise as a supplementary index for guiding functionally informed ablation therapy.

### 4.2 Sufficient Recording Duration for Reliable Estimation of Heterogeneous Conductions Using Phase Variance Analysis

We qualitatively and quantitatively evaluated the effect of analysis duration on the reliability of identifying heterogeneous conduction regions using phase variance analysis. Longer analysis durations improved similarity to the reference PV map generated from the full recording, while inter‐subsample variability decreased. With recordings of 10 s or longer, SSIM values consistently exceeded 0.95, even including outliers, and pixel‐wise standard deviation among 100 subsamples converged to < 0.015. Although strict thresholds for SSIM and standard deviation are difficult to define, the results shown in Figure 2 suggest that ~10 s of recording is sufficient to characterize conduction abnormalities with high accuracy and stability.

In clinical practice, longer recordings may improve accuracy but also extend procedure time, increasing patient and operator burden. Thus, a balance must be struck between diagnostic precision and procedural efficiency. Most conventional mapping techniques require several to tens of seconds of intracardiac ECG recording to reliably identify conduction abnormalities and arrhythmogenic substrates [15, 16]. Within this context, the 10‐s duration identified in our study appears clinically acceptable for achieving reliable substrate mapping with phase variance analysis.

### 4.3 Causes of Variability in Heterogeneous Conduction Estimation From Short‐Duration Recordings

Estimation of heterogeneous conduction regions from short‐duration recordings showed substantial variability across subsamplings, with localized abnormal activations disproportionately emphasized (Figure 2). This variability likely reflects the non‐periodic and complex activation patterns characteristic of persistent AF. Previous studies have also investigated the temporal stability of various substrate mapping techniques, including rotor mapping, dominant frequency analysis, and ExTRa Mapping. These studies consistently reported that, when the analysis was performed over relatively short recording durations such as several seconds, the estimated substrate or activation pattern exhibited notable temporal variability [17, 18], whereas extending the recording time improved the reproducibility and reliability of the results [19]. These findings are broadly consistent with our observations and support the necessity of sufficient recording duration to achieve stable characterization of conduction heterogeneity in persistent AF.

In theory, a single activation cycle should be sufficient for conduction pattern analysis under strictly periodic activation. However, our findings indicate that persistent AF requires substantially longer recordings than the typical cycle length. Longer recordings allow temporal averaging of transient phenomena such as conduction blocks or rotor activity, thereby providing a more accurate representation of the frequency and spatial distribution of abnormal activations.

### 4.4 Limitations

This study has several limitations. First, the analysis relied on membrane potential inference by DEAP Mapping [13], and estimation errors in this model may have affected the assessment of conduction heterogeneity. Although the accuracy of DEAP Mapping has been validated in ex vivo experiments, direct validation in the human atrium in vivo is not feasible due to the inability to obtain ground‐truth optical mapping data in clinical settings. Moreover, there are several potential differences between ex vivo and in vivo environments that could affect the accuracy of membrane potential estimation, including atrial wall thickness heterogeneity, far‐field ventricular signals, and catheter stability. In the present study, to mitigate these influences, catheter positioning was performed by experienced physicians who aimed to maintain a stable location during recordings, and post‐processing techniques were applied to suppress far‐field components. Nevertheless, these efforts may not have eliminated environmental differences, and we acknowledge this as a limitation of the study. Despite these limitations, the present study demonstrates the clinical applicability of phase variance analysis when combined with DEAP Mapping for estimating heterogeneous conduction regions from intracardiac ECGs.

Second, the reference PV maps were generated from 30‐s recordings. While longer recordings are generally assumed to be more reliable, they do not necessarily guarantee a complex reflection of the underlying activation dynamics. In clinical practice, longer recordings may be impractical due to increased procedure time and patient burden. For this retrospective analysis, we used the same 30‐s data employed for excitation mapping with the CARTO Finder. Importantly, the pixel‐wise standard deviation analysis across subsamples (Figure 3B), which is independent of the reference map, showed sufficient convergence at ~10 s. This supports the validity of the 30‐s recordings as a comparative baseline for assessing time dependency.

Third, the study included a limited number of patients. Although detailed intra‐patient subsample analysis was performed, the generalizability of our findings to the broader population of persistent AF patients remains to be validated. Future studies with larger cohorts are warranted to confirm the robustness and clinical utility of the proposed approach.

Finally, this study focused exclusively on visualizing heterogeneous conduction regions and did not assess the impact of phase variance analysis on ablation strategies or clinical outcomes. Although quantitatively identified regions of abnormal excitation may have potential utility in guiding ablation and improving outcomes, this hypothesis requires evaluation in future prospective clinical studies.

## 5 Conclusions

In this study, we investigated the influence of recording duration on the accuracy and reproducibility of estimating heterogeneous conduction regions using phase variance analysis in patients with persistent AF. Longer recordings increased similarity with the reference PV map and reduced variability across subsamples, both qualitatively and quantitatively. This improvement likely reflects temporal averaging of transient conduction changes, enabling more accurate representation of abnormal excitation frequency. These findings indicate that, even in persistent AF characterized by complex and nonperiodic conduction, phase variance analysis can provide reliable characterization of local activation heterogeneity when sufficient recording duration—approximately 10 s—is ensured.

## Funding

This work was supported by Japan Agency for Medical Research and Development (23he2822007j0001).

## Ethics Statement

The study was approved by the Institutional Review Board of The University of Tokyo Hospital (Approval No. 2023232NI) and conducted in accordance with the principles of the Declaration of Helsinki.

## Consent

An opt‐out consent process was implemented in accordance with institutional guidelines. Information about the study was provided to all eligible patients, and the opportunity to decline participation was offered.

## Conflicts of Interest

The authors declare no conflicts of interest.

## Data Availability

The data supporting the findings of this study are available from the corresponding author, N.T, upon reasonable request.
